# A novel Cys328-terminator mutant implicated in severe coagulation factor XIII deficiency: a case report

**DOI:** 10.1186/s12881-020-01111-0

**Published:** 2020-09-03

**Authors:** Ruimin Cai, Yi Li, Wenyang Wang, Qiang Feng

**Affiliations:** Department of Clinical Laboratory, Taian City Central Hospital, No. 29, Longtan Road, Taishan District, Taian, 271000 Shandong Province China

**Keywords:** Bleeding disorder, Factor XIII deficiency, Point mutation, A-subunit

## Abstract

**Background:**

Factor XIII (FXIII) deficiency is an extremely rare bleeding disorder that is commonly due to mutations in the FXIIIA subunit gene (F13A1), and it has been reported to have a prevalence of one per 2 million. We describe a new genetic variant in the F13A1 gene that caused a patient to suffer from lifelong hemorrhagic diathesis.

**Case presentation:**

We evaluated a 20-year-old female with umbilical cord bleeding after birth, an intracerebral hemorrhage at age 6, and other bleeding episodes, including hematuria and cephalohematoma, who suffered from a lifelong hemorrhagic diathesis. The clot solubility test showed that the clot of the patient was dissolved in urea solution at 10 h. Genetic testing identified a novel homozygous mutation, c.984C > A(p. Cys328stop), resulting in a premature stop codon in exon 8 of the F13A1 gene. The results obtained with ClusterX software showed that Cys328 of exon 8 in the F13A1 gene is highly conserved among species.

**Conclusion:**

We reported a novel homozygous mutation in the F13A1 gene in a factor XIII (FXIII)-deficient patient, which adds a new point mutation to the mutant library. In this paper, we discuss other aspects of the disease, including laboratory examination, homogeneous sequence alignment and molecular modeling.

## Background

Coagulation factor XIII (FXIII) is a plasma transglutaminase that is activated in the clotting cascade, which is essential for the final step in the blood coagulation process [1]. Plasma FXIII (pFXIII) is a tetramer composed of two A-subunits and two B-subunits. The A-subunits have catalytic function, and the B-subunits do not have enzymatic activity and may serve as plasma carrier molecules [2]. After cleavage of the peptide by thrombin in the presence of calcium ions, pFXIII dissociates from its B-subunits and yields the active enzyme factor XIIIa. This enzyme acts as a transglutaminase to catalyze the formation of γ-glutamyl-ε-lysine crosslinking between fibrin molecules, thus stabilizing the fibrin clot [3].

pFXIII A-subunits are expressed mainly in the placenta, urinary bladder, gall bladder and bone marrow; the pFXIII A-subunit gene consists of 15 exons, and the B-subunit gene is composed of 12 exons, while its expression is restricted to the liver. FXIII deficiency is classified into two categories: type I deficiency, which is characterized by the lack of both the A and B subunits; and type II deficiency, which is characterized by the lack of the A subunit alone. These defects can result in a lifelong bleeding tendency, defective wound healing, and habitual abortion and can even cause crippling bleeding or intracranial hemorrhage [4, 5].

FXIII A subunit deficiency has been reported with a prevalence of one per 2 million, while B subunit deficiency is even more rare. It has been reported that there are 203 mutations worldwide that have been identified in patients with FXIII deficiency, among which more than 90% are responsible for FXIII A deficiency. In addition to point mutations, there are also small deletions/insertions and even large deletions [6]. Developments in DNA technology and bioinformatics can help us to understand the structural and functional consequences of the various mutations of FXIII.

In this study, the clinical manifestations and laboratory results of FXIII-deficient patient are presented, and genetic analysis of F13A1 was performed by direct DNA sequencing. Furthermore, bioinformatics and homology analysis of the amino acid sequence were conducted. Our findings showed that the patient with severe FXIII deficiency was homozygous for a Cys328-terminator mutation in the A-subunit gene.

## Case presentation

The patient was a 20-year-old female. The first bleeding episode was umbilical cord bleeding after birth, and she was admitted to the hospital because of an intracerebral hemorrhage at the age of 6, and other bleeding episodes included hematuria and cephalohematoma. She suffered from swelling and ecchymosis around the thighs and ankle intermittently and consulted with doctors because of a lifelong hemorrhagic diathesis. The activated partial thromboplastin time (APTT), prothrombin time (PT), thrombin time (TT) and D-dimer were normal, fibrinogen was only slightly reduced to 1.97 g/L (normal range: 2 ~ 4 g/L), there was no family history of bleeding tendency, and informed consent was obtained from the patient.

A clot solubility test was conducted, and 0.1 ml plasma was collected from the patient. Then, 0.1 ml calcium chloride solution (0.025 mol/L) was added and mixed, and the sample was incubated for 1 h at 37 °C for clot formation. The clot was transferred into 5 mol/L urea solution, and then, the clot solution was regularly evaluated every 5 min for a total of 2 h and then every 2 ~ 4 h for a total of 24 h. According to the results of the clot solubility test, the urea solubility assay was positive, and the clot of the patient was dissolved at 10 h, which meant that the level of FXIII was approximately less than 5% [7].

**Fig. 1:**
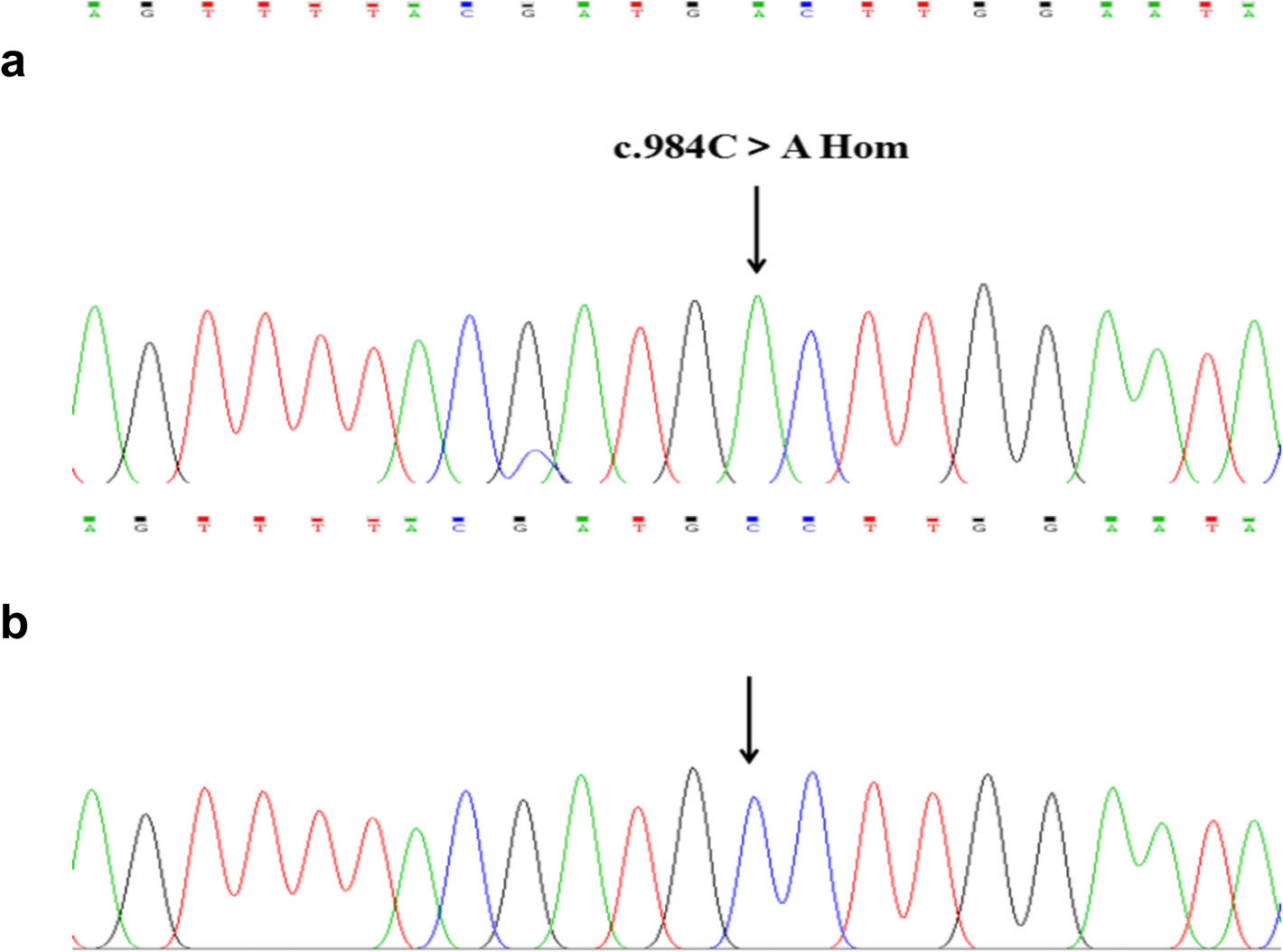
Identification of the mutation in F13A1 by DNA sequencing. c.984C > A was found in the patient (**a**); the GenBank sequence of F13A1 (**b**)

Genomic DNA was extracted from fresh blood samples using a DNA extraction kit (TIANGEN Biotech Co., Ltd., Beijing, China). All exons and flanking sequences of the FXIII A gene were amplified by polymerase chain reaction (PCR) using specific primers; the sequences of all the primers and PCR conditions were previously described in the literature [8]. DNA sequencing was performed by the Ruibiotech company (Beijing, China), and the sequencing results and corresponding GenBank sequence (AH002691.2) were analyzed by Megalign software to identify mutations. Genetic testing identified a novel homozygous mutation, c.984C > A, resulting in a premature stop codon in exon 8 of the F13A1 gene (p.Cys328stop) (Fig. 1), and there were no other mutations found in the exons of the F13A1 gene.

**Fig. 2:**
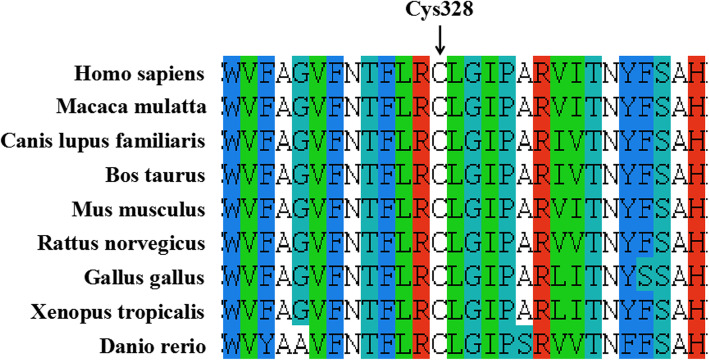
Homologous sequence alignment of Cys328 among different species

**Fig. 3:**
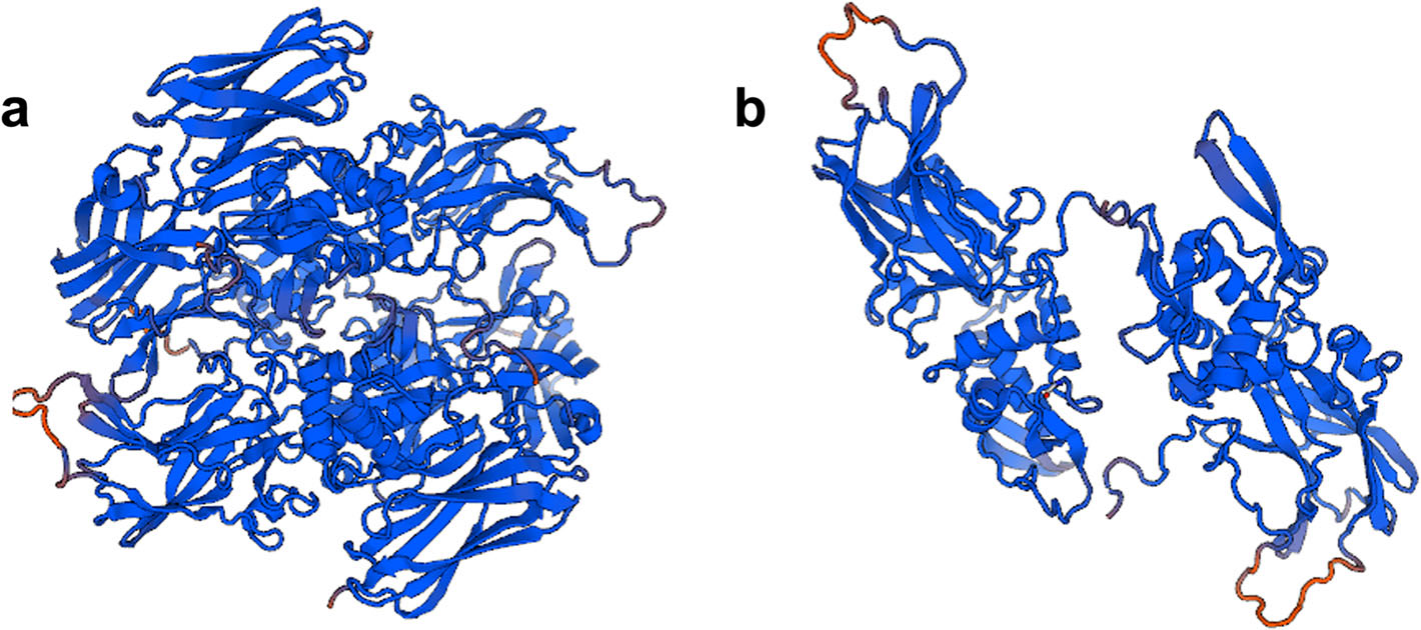
The three-dimensional structure of the FXIIIA subunit. **a**: the wild type; **b**: the mutant (p.Cys328stop). The three-dimensional structures of the wild-type and the mutant F13A1 protein generated by the SWISS-MODEL online software shows obvious differences, in which p.Cys328stop resulted in the termination of translation and the protein features were affected

Furthermore, p.Cys328stop was predicted to be disease-causing by MutationTaster. ClusterX software (version 1.81) was used to compare the sequence of F13A1 of humans (*Homo sapiens*, NP_000120.2) with that of different species, including *Macaca mulatta* (XP_001096779.2), *Canis lupus familiaris* (XP_535876.2), *Bos taurus* (NP_001161366.2), *Mus musculus* (NP_083060.2), *Rattus norvegicus* (NP_067730.2), *Gallus gallus* (NP_990016.1), *Xenopus tropicalis* (XP_002936471.1), and *Danio rerio* (NP_001070622.1). The results from ClusterX software showed that Cys328 in exon 8 of F13A1 is highly conserved among species (Fig. 2). The structure analysis of the FXIIIA molecule produced by the Swiss model is shown in Fig. 3.

## Discussion and conclusions

FXIII deficiency is a rare hemorrhagic disease, and defective FXIII A-subunits or B-subunits can cause FXIII deficiency, but the FXIIIA- subunit can hardly be detected in the plasma of patients with F13A1 gene deficiency. Although the FXIIIA-subunit in the plasma of patients with F13B gene deficiency is significantly reduced, it still remains active. In addition, 5% FXIII activity can maintain the normal hemostatic function of the body, so the population with a deficiency may not have obvious clinical characteristics; in some particular cases, it is easy to escape diagnosis or be misdiagnosed. As a result, gene sequencing improves the chances of diagnosis and treatment of FXIII deficiency.

Diagnosis of FXIII deficiency is still a challenge due to the normal routine coagulation tests, while the clot solubility assay has become the most widely used method for screening for FXIII deficiency. In our study, we used the urea solubility assay to preliminarily examine the patient. While the clot solubility assay is a qualitative test for the diagnosis of FXIII deficiency, there are some quantitative tests used in the laboratory diagnosis of FXIII deficiency, including activity and antigen assays; the former includes photometric assays, putrescine incorporation assays and fluorometric assays [3, 9]. Several assays can be used to measure the FXIII antigen, including immunoassays and a one-step sandwich ELISA method [10, 11].

According to the clinical symptoms, signs and routine laboratory test results, we found a patient with FXIII deficiency. The genetic defect type of the patient was determined to be a c.984C > A homozygous mutation (Cys328stop) in exon 8 of the FXIIIA subunit by genetic diagnosis, and Cys328 in exon 8 of F13A1 is highly conserved among different species. The mutation site in our study contained a nonsense mutation, which led to a premature stop codon, so it may produce a truncated harmful protein, which is a functional defective mutation.

## Data Availability

The reference sequence for validation of the Cys328-terminator variants in the F13A1 gene was acquired from the NCBI Nucleotide database by using accession number AH002691.2. and the reference sequence for different species of *Homo sapiens*, *Macaca mulatta*, *Canis lupus familiaris*, *Bos taurus*, *Mus musculus*, *Rattus norvegicus*, *Gallus gallus*, *Xenopus tropicalis* and *Danio rerio* was acquired from the NCBI Protein database by using accession number NP_000120.2, XP_001096779.2, XP_535876.2, NP_001161366.2, NP_083060.2, NP_067730.2, NP_990016.1, XP_002936471.1 and NP_001070622.1. The raw datasets are available from the corresponding author on reasonable request.

## Abbreviations

APTT: Activated partial thromboplastin time
FXIII: Factor XIII
PCR: Polymerase chain reaction
PT: Prothrombin time
TT: Thrombin time
